# Stories of strength: uncovering innate resilience in domestic violence survivors with ACEs scores of four or more

**DOI:** 10.3389/fpubh.2024.1352381

**Published:** 2024-03-20

**Authors:** Cassandra Velasco Adams, Barbara Gibson

**Affiliations:** Women's Resource Center to End Domestic Violence, Decatur, GA, United States

**Keywords:** Adverse Childhood Experiences (ACEs), domestic violence, intimate partner violence, community resiliency model, trauma informed approaches, resiliency focused care, recovery and healing, body based interventions

## Introduction

Adults and children accessing services with the Women's Resource Center safehouse have experienced deeply personal, complex, and often intersectional trauma. Displaced by domestic violence, many families participating in the emergency housing program have an Adverse Childhood Experiences (ACEs) score of four or more, based on the Adverse Childhood Experiences Study. Anecdotal evidence suggests that body-based, resiliency focused interventions with participating families can help mitigate the impact of ACEs.

*Body - and movement-oriented interventions can be defined as those in which physical activity and corporeality are the central themes and core focus of the intervention; they are characterized by their use of movement activities and focus on bodily experiences* *(**1**)*.

It is our privilege as advocates to first support survivors to stabilize from their figurative “critical condition,” to then begin to address the multiple compounding fractures underneath the presenting event of intimate partner violence. Deeply rooted trauma and the resulting biological and emotional responses can flatten capacity. This decreased ability to respond to daily demands sets a painful cycle of disappointment and self-mistrust in motion. Survivors often experience reduced capacity, or inability to act in their own best interests, as a personal failing rather than a response to trauma and ACEs.

Survivors may operate in survival mode even when danger is not looming. Survival responses impact emotion and behavior in ways that challenge participants' ability to plan and attend to tasks that support stability and wellbeing over the long term. Ironically, operating in survival mode can slow or stop actions that transform surviving to thriving.

During the months they call the safehouse home, families and advocates work together to begin reconstructing the foundation for safe and stable lives. Essential building blocks include employment, childcare, housing, and transportation. These depend on a gradually strengthening foundation of physical, mental, and emotional health for greater stability over the long-term. Families continue this process for many more months as they access transition support. Resiliency-focused interventions can help survivors better relate to and manage the experience of somatic distress,^1^ thus increasing capacity to attend to activities of daily living that bolster stability.

Domestic Violence is a public health crisis tangled in a web of complex traumas with deeply personal, systemic, and societal consequences. The risk factors or red flags for domestic violence are well researched (2). But what are best practices in the aftermath of trauma and violence? This paper offers examples based on our support of domestic violence survivors with ACEs scores of four or more. It is important to understand that problematic responses to trauma (3) don't happen because people are bad, don't care or are lazy, they happen because trauma dysregulates.

Dysregulation can negatively impact feelings and behavior to the degree that even when motivation is high, capacity for action may be low or absent. This is important to name because it helps survivors regard their own experiences with more compassion and less shame. Body-based, resiliency-focused interventions offer ways of coping that nurture wellbeing and pave new pathways to regulation. These new pathways work with, rather than against, survivors' hopes for stability.

Though there have been studies highlighting the scientific connection to using secular mindfulness with an increase in overall mind-body wellbeing for the general public (4),^2^ there have not been many studies investigating mindfulness techniques and compassion training to increase resilience in the sub-population of intimate partner/domestic violence survivors. WRC has adapted trauma-informed practices, including mindfulness techniques through experiential knowledge, into a sort of “trauma intensive care program” designed to cultivate resilience, foster self-regulation, practice positive coping skills, and establish long-term stability, safety, and self-care.

Our experiences have demonstrated how much mind-body wellness impacts individual progress toward positive future building. Toxic stress dysregulates the nervous system. Individuals then have less ability to marshal the inner resources needed to align daily tasks with deeply held values and goals.

We see survivors navigating daily life triggered and retriggered into fight or flight responses, essentially pushing their bodies into automated survival responses that bypass the logical brain and exhaust the emotional one. This state of dysregulation interrupts their ability to manage reactivity and, therefore, to make peaceful or sound decisions with long term consequences in mind.

Along with dysregulated states, we have observed hypervigilance in survivors. As expected, they struggle to tap into any felt sense of safety and are thus hyper attuned to cues of real *and* perceived danger around them with reduced ability to recognize cues of safety. This is how Debra felt. Debra had experienced a lifetime of trauma before coming into the safehouse with her children. Her mom struggled through substance use disorder and died when Debra was a teenager. Debra had a chaotic childhood and became justice involved as a young adult. Domestic violence, housing and income instability compounded her trauma. Safe and stably housed for more than one year now, Debra counts peer support as one of the most important parts of her healing - “*I felt like my feelings were valid and accepted. You sat with me in the storm, you did not try to rush me through my feelings. You accepted my feelings and encouraged me to have compassion for myself and be gentle with my feelings because they make sense with everything I have gone through. It was weird but I felt safe. That felt like a turning point.”* This kind of affirming, embodied witnessing with survivors, of opening space to experience the feeling of being seen and deeply listened to can be very healing.

Helen similarly shared, “*when I came to this house my mind wasn't working. Yoga and sound bowl meditation helped me settle, and I was able to think about what I needed to do for myself and my girls and actually do it.”* Helen learned to identify and respond to uncomfortable sensations in her body. Practicing yoga gave her more confidence to manage dysregulation and not have it hijack her emotions or her plans all the time. Helen describes now being better able to recognize and respond to the bodily sensations associated with distress and overwhelm.

At our safehouse, families participate in trauma-informed, resiliency-focused intervention programs to support just this type of healing. These include weekly talk therapy sessions, yoga, parenting groups, meditation and financial literacy groups, art therapy, social, emotional, ethical (SEE)^3^ learning, and the Community Resiliency Model (5) (CRM). Our intention in providing these services is to normalize nervous system regulation *and* dysregulation. This is important because survivors often have the sense that something is wrong with them or that they are getting something wrong when they experience dysregulation. They aren't. We all feel dysregulated sometimes. Also important to our work is engaging survivors in practices that bring attention to experiences of regulation. Even brief moments of feeling ease or safety in the body can serve as a reminder that dysregulation need not be a permanent or default state. There is hopefulness here, a good place to confidently embark on future planning.

Preliminary evidence^4^ from a ten participant focus group points to improved physical and emotional health, reduced somatic and emotional distress, greater learning, and school engagement among children, as well as increased self-efficacy with decreased use of maladaptive coping practices among children and adults. These trauma-informed, resiliency-focused programs help survivors touch into a sense of peace in their bodies as well as open to possibility in their minds. In time, we believe this will foster self-compassion and nervous system healing that transcends the weight of trauma, shame, and grief.

These interventions continue, if families choose, after transition from the safehouse. For families this happens with Women Moving On transition support, and for adults in the SISTERS group (Survivors in Service To Extend Resiliency) with ongoing wellbeing workshops and activities such as volunteer, exercise, and discussion groups. Feeling like you belong matters, and reliable, healthy connections can be healing. We also have an 8-week summer camp (Camp PEACE), free for families, that brings school-aged children exposed to domestic violence together for trauma-informed, peace education, SEE learning, future building, and fun.

Individuals and families using these interventions are growing incrementally stronger, despite complex trauma. We attribute this to increasing confidence in their ability to skillfully manage painful thoughts, sensations, and feelings. Survivors with ACEs scores of four or more need not be trapped in dysregulated states or limited by the past. Trauma-informed, resiliency-focused support provides important interventions that aid survivors in rebuilding safe and stable lives. Our hoped for outcome is ongoing healing, health, and happiness despite adversity. It is through body-based, resiliency-focused interventions, community building, and safe spaces to practice positive coping skills that we have seen survivors grow toward these deeply deserved outcomes.

## Author contributions

CV: Writing – original draft. BG: Writing – original draft.

## Footnotes

^1^Perishable, “SE 101.”

^2^“What Are the Benefits of Mindfulness?”

^3^*See learning - home*. https://seelearning.emory.edu/en/home.

^4^Formal evaluation is planned to begin in May 2024 with support from an MPH fellow.

## References

[1] van de Kamp MM Scheffers M Hatzmann J Emck C Cuijpers P Beek PJ. Body- and movement-oriented interventions for posttraumatic stress disorder: a systematic review and meta-analysis. J Trauma Stress. (2019) 32:967–76. 10.1002/jts.22465 31658401 PMC6973294

[2] Capaldi DM Knoble NB Shortt JW Kim HK. A systematic review of risk factors for intimate partner violence. Partner Abuse. (2012) 3:231–80. 10.1891/1946-6560.3.2.231 22754606 PMC3384540

[3] Center for Substance Abuse Treatment (US). Understanding the impact of trauma. In: Trauma-Informed Care in Behavioral Health Services. Rockville (MD): Substance Abuse and Mental Health Services Administration (2014). 24901203

[4] Schreiner I Malcolm JP. The Benefits of Mindfulness Meditation: Changes in Emotional States of Depression, Anxiety, and Stress. Cambridge, MA: Cambridge University Pres (2012).

[5] Miller-Karas E. Building Resilience to Trauma: The Trauma and Community Resiliency Models. Routledge: Taylor & Francis Group (2023).

